# 3-[3-(2-Pyrid­yl)-1*H*-pyrazol-1-yl]propan­amide

**DOI:** 10.1107/S1600536809019060

**Published:** 2009-05-29

**Authors:** Feng Huang, Bin Jin, Jian-Feng Zhang

**Affiliations:** a State Key Laboratory Base of Novel Functional Materials and Preparation Science, Faculty of Materials Science and Chemical Engineering, Ningbo University, Ningbo, Zhejiang 315211, People’s Republic of China

## Abstract

In the title compound, C_11_H_12_N_4_O, the pyrazole and pyridine rings are nearly coplanar [dihedral angle = 1.87 (5)°]. Adjacent mol­ecules are linked by N—H⋯N and N—H⋯O hydrogen bonds into a linear chain running along the *c* axis.

## Related literature

For the chemistry of 3-(2-pyrid­yl)pyrazoles, see: Ruben *et al.* (2004 ▶); Steel (2005 ▶).
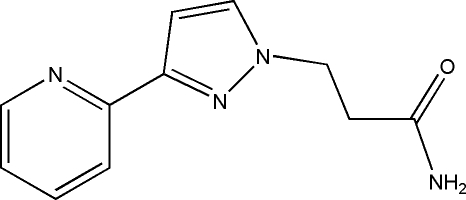


## Experimental

### 

#### Crystal data

C_11_H_12_N_4_O
*M* *_r_* = 216.25
Triclinic, 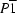

*a* = 7.7446 (15) Å
*b* = 8.3517 (17) Å
*c* = 8.4804 (17) Å
α = 97.99 (3)°
β = 98.95 (3)°
γ = 90.40 (3)°
*V* = 536.4 (2) Å^3^
*Z* = 2
Mo *K*α radiation
μ = 0.09 mm^−1^
*T* = 293 K
0.58 × 0.55 × 0.27 mm

#### Data collection

Rigaku R-AXIS RAPID diffractometer
Absorption correction: multi-scan (*ABSCOR*; Higashi, 1995 ▶) *T*_min_ = 0.947, *T*_max_ = 0.972
5019 measured reflections
2410 independent reflections
1937 reflections with *I* > 2σ(*I*)
*R*_int_ = 0.029

#### Refinement

*R*[*F* ^2^ > 2σ(*F* ^2^)] = 0.045
*wR*(*F* ^2^) = 0.139
*S* = 1.12
2410 reflections
146 parameters
H-atom parameters constrained
Δρ_max_ = 0.37 e Å^−3^
Δρ_min_ = −0.28 e Å^−3^

### 

Data collection: *RAPID-AUTO* (Rigaku, 1998 ▶); cell refinement: *RAPID-AUTO*; data reduction: *CrystalStructure* (Rigaku/MSC, 2004 ▶); program(s) used to solve structure: *SHELXS97* (Sheldrick, 2008 ▶); program(s) used to refine structure: *SHELXL97* (Sheldrick, 2008 ▶); molecular graphics: *SHELXTL* (Sheldrick, 2008 ▶); software used to prepare material for publication: *SHELXL97*.

## Supplementary Material

Crystal structure: contains datablocks global, I. DOI: 10.1107/S1600536809019060/ng2581sup1.cif

Structure factors: contains datablocks I. DOI: 10.1107/S1600536809019060/ng2581Isup2.hkl

Additional supplementary materials:  crystallographic information; 3D view; checkCIF report

## Figures and Tables

**Table 1:** Hydrogen-bond geometry (Å, °)

*D*—H⋯*A*	*D*—H	H⋯*A*	*D*⋯*A*	*D*—H⋯*A*
N1—H1*A*⋯O1^i^	0.86	2.11	2.968 (2)	175
N1—H1*B*⋯N4^ii^	0.86	2.21	3.055 (2)	167

Symmetry codes: (i) 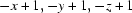; (ii) 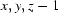.

**Fig. 1.**
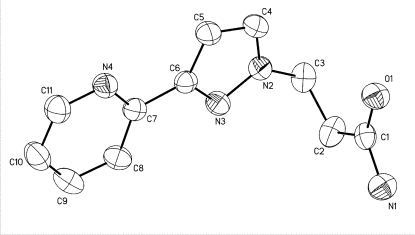
The molecular structure of the title compound. Displacement ellipsoids are drawn at the 45% probability level.

**Fig. 2.**
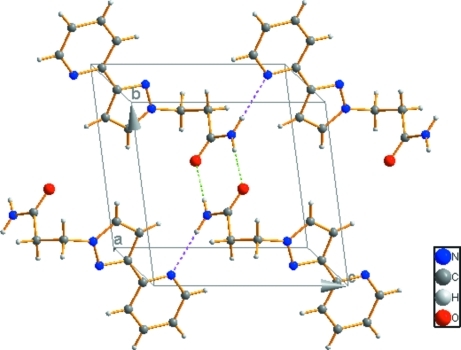
Partial packing view of the title compound. Dashed lines indicate N—H···N and N—H···O hydrogen bonds.

## supplementary crystallographic information

### Comment

Great attention has been paid to 3-(2-pyridyl)pyrazole-based ligands in the area of coordination chemistry, not only due to they can act as bridging or chelate ligands and their intriguing structures, but also for their potential applications as functional materials (Ruben *et al.*, 2004; Steel *et al.*, 2005). Herein, We report the structure of a *N*-Pyrazolylpropanamide ligand, C_11_H_12_N_4_O (Scheme 1).

As is shown in Figure 1, in the title compound, the dihedral angle between pyrazole and pyridine ring is 1.87 (5)°, and the torsion angle of N3—C6—C7—N4 is 179.36 (2)°. The molecules are formed into a three-dimensional supermolecular network through intermolecular weak N—H···N (N···N= 3.055 (2) Å) and N—H···O (N···O= 2.968 (2) Å) hydrogen bonds (Figure 2).The hydrogen bond geometry parameters are list in Table 1. Weak π-π stacking interactions between pyrazole ring (N2/N3/C6/C5/C4) and pyridine ring (N4/C7/C8/C9/C10/C11) (symmetric code: -*x*, -*y*, 2 - *z*), with a centroid-to-centroid distance of 3.828 (1)Å and interplanar distance of 3.739 (1) Å, help to stabilize the crystal structure.

### Experimental

A mixture of 3-(2-pyridyl)pyrazole (2.9 g, 20 mmol), sodium hydroxide (0.16 g, 4 mmol), *N*,*N*'-dimethylformamide(DMF)(100 ml) was stirred and heated to 373 k. A solution of acrylamide (1.44 g, 20 mmol) solubilized in DMF(10 ml)was added dropwise over a period of 10 minutes. After 7 h, heating was then terminated, and the solution was cooled to room temperature. The mixture was filtered, and DMF was removed by vacuum distillation. The product was then recrystallized from ethanol (yield: 64.7%; mp: 427 K). Calculated for C_11_H_12_N_4_O: C 61.10, H 5.59, N 25.91%; found: C 60.03, H 5.48, N 25.86%.

### Refinement

H atoms bound to C and N atoms were positioned geometrically and treated in the subsequent refinement as riding atoms, with C—H = 0.93 (aromatic) or 0.97 Å (methylene) and N—H = 0.86 Å, and with *U*_iso_(H) = 1.2 *U*_eq_(C,*N*).

### Crystal data

**Table:**

C_11_H_12_N_4_O	*Z* = 2
*M**_r_* = 216.25	*F*(000) = 228
Triclinic, *P*1	*D*_x_ = 1.339 Mg m^−^^3^
Hall symbol: -P 1	Mo *K*α radiation, λ = 0.71073 Å
*a* = 7.7446 (15) Å	Cell parameters from 5019 reflections
*b* = 8.3517 (17) Å	θ = 3.2–27.4°
*c* = 8.4804 (17) Å	µ = 0.09 mm^−^^1^
α = 97.99 (3)°	*T* = 293 K
β = 98.95 (3)°	Block, colorless
γ = 90.40 (3)°	0.58 × 0.55 × 0.27 mm
*V* = 536.4 (2) Å^3^	

### Data collection

**Table:**

Rigaku R-AXIS RAPID diffractometer	2410 independent reflections
Radiation source: fine-focus sealed tube	1937 reflections with *I* > 2σ(*I*)
graphite	*R*_int_ = 0.029
Detector resolution: 0 pixels mm^-1^	θ_max_ = 27.4°, θ_min_ = 3.2°
ω scans	*h* = −9→9
Absorption correction: multi-scan (*ABSCOR*; Higashi, 1995)	*k* = −10→10
*T*_min_ = 0.947, *T*_max_ = 0.972	*l* = −10→10
5019 measured reflections	

### Refinement

**Table:**

Refinement on *F*^2^	Secondary atom site location: difference Fourier map
Least-squares matrix: full	Hydrogen site location: inferred from neighbouring sites
*R*[*F*^2^ > 2σ(*F*^2^)] = 0.045	H-atom parameters constrained
*wR*(*F*^2^) = 0.139	*w* = 1/[σ^2^(*F*_o_^2^) + (0.0807*P*)^2^ + 0.0265*P*] where *P* = (*F*_o_^2^ + 2*F*_c_^2^)/3
*S* = 1.12	(Δ/σ)_max_ < 0.001
2410 reflections	Δρ_max_ = 0.37 e Å^−^^3^
146 parameters	Δρ_min_ = −0.28 e Å^−^^3^
0 restraints	Extinction correction: *SHELXL97* (Sheldrick, 2008), Fc^*^=kFc[1+0.001xFc^2^λ^3^/sin(2θ)]^-1/4^
Primary atom site location: structure-invariant direct methods	Extinction coefficient: 0.038 (11)

### Special details

**Table:**

Geometry. All e.s.d.'s (except the e.s.d. in the dihedral angle between two l.s. planes) are estimated using the full covariance matrix. The cell e.s.d.'s are taken into account individually in the estimation of e.s.d.'s in distances, angles and torsion angles; correlations between e.s.d.'s in cell parameters are only used when they are defined by crystal symmetry. An approximate (isotropic) treatment of cell e.s.d.'s is used for estimating e.s.d.'s involving l.s. planes.
Refinement. Refinement of *F*^2^ against ALL reflections. The weighted *R*-factor *wR* and goodness of fit *S* are based on *F*^2^, conventional *R*-factors *R* are based on *F*, with *F* set to zero for negative *F*^2^. The threshold expression of *F*^2^ > σ(*F*^2^) is used only for calculating *R*-factors(gt) *etc*. and is not relevant to the choice of reflections for refinement. *R*-factors based on *F*^2^ are statistically about twice as large as those based on *F*, and *R*- factors based on ALL data will be even larger.

### Fractional atomic coordinates and isotropic or equivalent isotropic displacement parameters (Å^2^)

**Table:**

	*x*	*y*	*z*	*U*_iso_*/*U*_eq_	
N1	0.41230 (16)	0.30020 (14)	0.39463 (14)	0.0502 (3)	
H1A	0.5037	0.3623	0.4058	0.060*	
H1B	0.4039	0.2110	0.3293	0.060*	
O1	0.28772 (12)	0.46829 (11)	0.57395 (12)	0.0495 (3)	
C1	0.28366 (17)	0.34222 (15)	0.47849 (14)	0.0391 (3)	
C2	0.13022 (19)	0.22241 (18)	0.44677 (16)	0.0509 (4)	
H2A	0.0683	0.2257	0.3387	0.061*	
H2B	0.1748	0.1145	0.4503	0.061*	
C3	0.00195 (18)	0.25207 (18)	0.56416 (17)	0.0477 (4)	
H3A	−0.0373	0.3623	0.5664	0.057*	
H3B	−0.0994	0.1801	0.5266	0.057*	
N2	0.07610 (14)	0.22676 (13)	0.72718 (13)	0.0403 (3)	
C4	0.0879 (2)	0.33312 (17)	0.86282 (18)	0.0500 (4)	
H4A	0.0518	0.4395	0.8703	0.060*	
C5	0.1622 (2)	0.25694 (17)	0.98736 (17)	0.0496 (4)	
H5A	0.1874	0.2996	1.0959	0.060*	
C6	0.19232 (15)	0.09985 (15)	0.91577 (15)	0.0367 (3)	
N3	0.13956 (14)	0.08220 (13)	0.75618 (13)	0.0401 (3)	
C7	0.26942 (15)	−0.03550 (14)	0.99317 (15)	0.0368 (3)	
C8	0.28816 (18)	−0.18581 (16)	0.90392 (18)	0.0457 (3)	
H8A	0.2547	−0.2021	0.7926	0.055*	
C9	0.3569 (2)	−0.30988 (17)	0.9829 (2)	0.0555 (4)	
H9A	0.3706	−0.4110	0.9253	0.067*	
C10	0.4054 (2)	−0.28298 (19)	1.1479 (2)	0.0576 (4)	
H10A	0.4498	−0.3655	1.2041	0.069*	
C11	0.3862 (2)	−0.13031 (19)	1.22693 (19)	0.0538 (4)	
H11A	0.4210	−0.1116	1.3381	0.065*	
N4	0.32045 (15)	−0.00708 (14)	1.15354 (14)	0.0451 (3)	

### Atomic displacement parameters (Å^2^)

**Table:**

	*U*^11^	*U*^22^	*U*^33^	*U*^12^	*U*^13^	*U*^23^
N1	0.0535 (7)	0.0437 (6)	0.0500 (7)	−0.0114 (5)	0.0099 (5)	−0.0069 (5)
O1	0.0528 (6)	0.0395 (5)	0.0531 (6)	−0.0070 (4)	0.0085 (5)	−0.0038 (4)
C1	0.0461 (7)	0.0361 (6)	0.0327 (6)	−0.0044 (5)	−0.0025 (5)	0.0070 (5)
C2	0.0590 (9)	0.0539 (8)	0.0366 (7)	−0.0193 (7)	0.0031 (6)	0.0012 (6)
C3	0.0437 (7)	0.0515 (8)	0.0466 (8)	−0.0067 (6)	−0.0011 (6)	0.0121 (6)
N2	0.0428 (6)	0.0378 (6)	0.0409 (6)	0.0012 (4)	0.0078 (4)	0.0057 (4)
C4	0.0662 (9)	0.0374 (7)	0.0486 (8)	0.0102 (6)	0.0171 (7)	0.0040 (6)
C5	0.0715 (9)	0.0410 (7)	0.0364 (7)	0.0097 (6)	0.0124 (6)	0.0007 (5)
C6	0.0361 (6)	0.0354 (6)	0.0389 (7)	−0.0020 (5)	0.0092 (5)	0.0028 (5)
N3	0.0408 (6)	0.0356 (5)	0.0421 (6)	−0.0008 (4)	0.0037 (4)	0.0019 (4)
C7	0.0326 (6)	0.0358 (6)	0.0418 (7)	−0.0022 (5)	0.0071 (5)	0.0031 (5)
C8	0.0439 (7)	0.0412 (7)	0.0479 (8)	0.0025 (5)	0.0031 (6)	−0.0025 (6)
C9	0.0539 (8)	0.0384 (7)	0.0704 (10)	0.0086 (6)	0.0045 (7)	−0.0001 (7)
C10	0.0580 (9)	0.0467 (8)	0.0698 (11)	0.0122 (7)	0.0072 (7)	0.0175 (7)
C11	0.0603 (9)	0.0545 (8)	0.0469 (8)	0.0079 (7)	0.0048 (6)	0.0120 (7)
N4	0.0515 (7)	0.0417 (6)	0.0417 (6)	0.0033 (5)	0.0072 (5)	0.0041 (5)

### Geometric parameters (Å, °)

**Table:**

N1—C1	1.3332 (18)	C5—C6	1.4040 (18)
N1—H1A	0.8600	C5—H5A	0.9300
N1—H1B	0.8600	C6—N3	1.3386 (16)
O1—C1	1.2328 (16)	C6—C7	1.4693 (18)
C1—C2	1.5150 (18)	C7—N4	1.3431 (18)
C2—C3	1.511 (2)	C7—C8	1.3938 (19)
C2—H2A	0.9700	C8—C9	1.377 (2)
C2—H2B	0.9700	C8—H8A	0.9300
C3—N2	1.4566 (18)	C9—C10	1.378 (2)
C3—H3A	0.9700	C9—H9A	0.9300
C3—H3B	0.9700	C10—C11	1.376 (2)
N2—C4	1.3427 (19)	C10—H10A	0.9300
N2—N3	1.3464 (16)	C11—N4	1.3389 (19)
C4—C5	1.362 (2)	C11—H11A	0.9300
C4—H4A	0.9300		
			
C1—N1—H1A	120.0	C4—C5—C6	104.82 (13)
C1—N1—H1B	120.0	C4—C5—H5A	127.6
H1A—N1—H1B	120.0	C6—C5—H5A	127.6
O1—C1—N1	123.19 (12)	N3—C6—C5	110.81 (12)
O1—C1—C2	122.26 (12)	N3—C6—C7	120.56 (11)
N1—C1—C2	114.55 (11)	C5—C6—C7	128.63 (12)
C3—C2—C1	114.53 (11)	C6—N3—N2	104.84 (10)
C3—C2—H2A	108.6	N4—C7—C8	121.89 (13)
C1—C2—H2A	108.6	N4—C7—C6	116.74 (11)
C3—C2—H2B	108.6	C8—C7—C6	121.38 (12)
C1—C2—H2B	108.6	C9—C8—C7	119.08 (14)
H2A—C2—H2B	107.6	C9—C8—H8A	120.5
N2—C3—C2	112.93 (12)	C7—C8—H8A	120.5
N2—C3—H3A	109.0	C8—C9—C10	119.41 (14)
C2—C3—H3A	109.0	C8—C9—H9A	120.3
N2—C3—H3B	109.0	C10—C9—H9A	120.3
C2—C3—H3B	109.0	C11—C10—C9	118.01 (15)
H3A—C3—H3B	107.8	C11—C10—H10A	121.0
C4—N2—N3	111.95 (11)	C9—C10—H10A	121.0
C4—N2—C3	127.56 (12)	N4—C11—C10	123.96 (15)
N3—N2—C3	120.47 (11)	N4—C11—H11A	118.0
N2—C4—C5	107.59 (13)	C10—C11—H11A	118.0
N2—C4—H4A	126.2	C11—N4—C7	117.61 (12)
C5—C4—H4A	126.2		

### Hydrogen-bond geometry (Å, °)

**Table:**

*D*—H···*A*	*D*—H	H···*A*	*D*···*A*	*D*—H···*A*
N1—H1A···O1^i^	0.86	2.11	2.968 (2)	175
N1—H1B···N4^ii^	0.86	2.21	3.055 (2)	167

Symmetry codes: (i) −*x*+1, −*y*+1, −*z*+1; (ii) *x*, *y*, *z*−1.
